# A forecast model for prevention of foodborne outbreaks of non-typhoidal salmonellosis

**DOI:** 10.7717/peerj.10009

**Published:** 2020-11-10

**Authors:** Fernando Rojas, Claudia Ibacache-Quiroga

**Affiliations:** 1 Centro de Micro-Bio Innovación, Universidad de Valparaíso, Valparaíso, Chile; 2 Escuela de Nutrición y Dietética, Facultad de Farmacia, Universidad de Valparaíso, Valparaíso, Chile

**Keywords:** Forecast, GSARIMA model, Salmonella outbreaks, Surveillance

## Abstract

**Background:**

This work presents a forecast model for non-typhoidal salmonellosis outbreaks.

**Method:**

This forecast model is based on fitted values of multivariate regression time series that consider diagnosis and estimation of different parameters, through a very flexible statistical treatment called generalized auto-regressive and moving average models (GSARIMA).

**Results:**

The forecast model was validated by analyzing the cases of *Salmonella enterica* serovar Enteritidis in Sydney Australia (2014–2016), the environmental conditions and the consumption of high-risk food as predictive variables.

**Conclusions:**

The prediction of cases of *Salmonella enterica* serovar Enteritidis infections are included in a forecast model based on fitted values of time series modeled by GSARIMA, for an early alert of future outbreaks caused by this pathogen, and associated to high-risk food. In this context, the decision makers in the epidemiology field can led to preventive actions using the proposed model.

## Introduction and Bibliographical review

Non-typhoidal salmonellosis is a foodborne illness considered as a major health issue with a great impact on the economy and the food industry. This diarrhea-producing pathology is globally distributed, with 93 million cases worldwide and 155,000 deaths per year (Majowicz et al., 2010). The main challenges for the control of this pathology are the environmental ubiquity of the pathogenic agents, their spreading pathways and their presence in food (Hamlet et al., 2006). Yerushalmy & Palmer (1959) proposed the term epidemetrics, which emphasized the quantitative nature of epidemiologic studies. In this scenario, new models for the accurate prediction of infectious outbreaks are needed. Brauer, Castillo-Chavez & Feng (2019) focused on the development of these models and highlighted the need to update them according to the advances in statistical science. Currently, a new generation of surveillance strategies have emerged, whose help to detect emerging infections and identify a high risk of outbreaks of infectious diseases, related to climate change and other factors. Traditional surveillance methods are based on retrospective strategies, therefore the development of new epidemiological models that allow the prediction of infectious outbreaks is of great interest, in order to take the necessary measures to limit their expansion and impact on public health (Rees et al., 2019). In these types of methods, risk factors (explanatory variables, such as open source internet data) are used to predict the outcome of interest (for example, number of cases reported) (Ginsberg et al., 2009). For example, Santillana et al. (2014) used these types of regression models to forecast the number of cases of seasonal influenza in the future. In this context, regression methods can be expanded using machine learning algorithms, to find complex associations between the result and the explanatory variables (Santillana et al., 2015).

Many of the current epidemetric models, like simulation, were implemented several years ago and need to be updated (Mori, 1996). In this scenario, the control of non-typhoidal salmonellosis should include new innovative surveillance and forecast statistical tools for its prevention, specially focused on the production and consumption of high-risk food (Boyen et al., 2008; Ashton et al., 2016) and improving the existing predicting tools (Thakur & Anbanandam, 2017). Non-typhoid salmonellosis outbreaks have been widely associated to climatic and environmental factors, like extreme rainfall, flooding, increased average temperatures, augmented frequency of extreme high-temperatures and changes in weather seasonal patterns (Amuakwa-Mensah, Marbuah & Mubanga, 2017). In this context, these parameters should be considered for the development of new models.

One of the strategies used to evaluate the link between number of infections and predictive variables (co-variables) for the monitoring, prediction and measurement of the impact of interventions is the analysis of time series, nevertheless, the vast majority of the approximations using Gaussian methods are prone to inaccuracies when the case counts are low and the bias of the statistical distribution of the real data is not symmetric (Allen, 2017). Therefore, appropriate statistical methods are required for count data, which allows the identification of infectious outbreaks through high precision forecasting.

Often, the count of reports of infections is a random variable that depends on time (Konradsen et al., 2000). In order to adequately describe this variable, the modeling of the possible temporal dependence related to multiple periods is needed. This time dependence of a variable can be described by an autoregressive moving average (ARMA) model in a time series.

The advantage of considering an ARMA framework lies in its malleability to model time-dependent variables, its easy estimation and interpretation, and its prediction power (Gilbert, 2005). On the other hand, its main disadvantages are related to its linear formulation between the response indexed in the time and its predictors, like the assumption that the modeled variable follows a Gaussian probability distribution. To overcome this last restriction, the original data are transformed, generating a complication in the interpretation of results (Fischer & Kamps, 2011).

To improve these limitations, McCullagh & Nelder (1983) formulated generalized linear models (GLM). In these models, the error is not modeled like in the classic regression models, but it is assumed that the response variable can be modeled by some type of statistical distribution belonging to the exponential family, where the normal distribution is a particular case. This approach allows linear and non-linear linking of the mean of the statistical distribution that best fits the real data of the response variable, respect the predictors through a linking function. In this context, Benjamin, Rigby & Stasinopoulos (2003) proposed a GLM version of ARMA models known as Generalized ARMA (GLARMA). In this model, the systematic component allows us to express a function of the mean (the link function) by means of an additive arrangement, with parameters or coefficients that indicate the direction and magnitude of the relationship with explanatory variables (covariates) and the autoregressive and moving average components. This type of formulation gains in flexibility and the possibility of using other types of non-linear associations, under the ARMA framework. The parameters of a GLARMA model can be estimated using the maximum likelihood (ML) method, assuming a statistical distribution of the exponential family for the response variable. Often, a normal (or Gaussian) distribution is considered in the modeling of random variables (Box et al., 2015), but other distributions might also be assumed (Rojas, 2016; Rojas et al., 2019). ARMA and GLARMA models are often used to predict future values (Benjamin, Rigby & Stasinopoulos, 2003; Calfa, 2015). GLARMA models are also used to estimate mean values and find the conditional probability density function (PDF) to past data, like what occurs with random variables when temporal dependence and covariates are present. This last aspect is of particular interest in stochastic predictive models.

If the time series for modeling is non stationary and/or a stochastic seasonal component is considered, the GLARMA model described by Benjamin, Rigby & Stasinopoulos (2003) is appropriate. To overcome the aforementioned restrictions, GLARMA evolved into the generalized multiplicative seasonal autoregressive integrated moving average (GSARIMA) models, considering the differentiation and seasonality components in its formulation, see Briët, Amerasinghe & Vounatsou (2013).

The aim of this paper is to propose a forecast model based on fitted values of multivariate time series and its projection, for prevention of non-typhoidal salmonellosis outbreaks, considering diagnosis and estimation of parameters obtained from GSARIMA model, given the high predicted accuracy that this model can achieve, which makes it a useful tool for epidemiological prevention.

## Methodology

### GLM, GLARMA and GSARIMA framework

Let *Y* be an RV related to the counting reports of infections by a pathogen of interest such as **Salmonella enterica**. We consider that statistical distribution of *Y* it belongs to the exponential family and that its PDF is

(1) \begin{eqnarray*}{f}_{Y}(y;\vartheta ,\varphi )=\exp \nolimits \left( \frac{y\vartheta -b(\vartheta )}{\varphi } +c(y,\varphi ) \right) , y\in {R}_{Y},\end{eqnarray*}

where *ϑ*, *φ* are canonical and scale parameters, respectively, *R*_*Y*_ is the support of *Y* and *b*, *c* are specific functions whose characterize a member of the exponential family of statistical distributions.

This parametrization has the following properties for the description of mean and variance as the first and second derivatives of the canonical and scale parameters, where E(*Y*) = *b*′(*ϑ*) and Var(*Y*) = *φb*″(*ϑ*), respectively.

In GLM, a function denominated link function of the mean of *Y* is equal to a systemic component, then *g*(*μ*) = *η*. In turn, the mean of *Y* corresponds to the inverse link function of *η*, which is related to know values of *r* covariates ***x*** = (***x***_0_, ***x***_1_, …, ***x***_*r*_)^⊤^, with ***x***_0_ = 1, where

(2) \begin{eqnarray*}\mu =\text{E}(Y)={g}^{-1}(\eta )={g}^{-1}({\mathbi{x}}^{\top }\beta ),\end{eqnarray*}

with *β* = (*β*_0_, *β*_1_, …, *β*_*r*_)^⊤^ being the regressors associated with ***x***.

Now we will derive the expressions for the case of an RV as *Y* but ordered in a temporal sequence *t*, this RV indexed over time *t*, with *t* = 1, …, *n* is denominated *Y*_*t*_. The conditional distribution of *Y*_*t*_ given the past data set

(3) \begin{eqnarray*}{\mathbi{H}}_{t}=\{{\mathbi{x}}_{1},\ldots ,{\mathbi{x}}_{t},{y}_{1},\ldots ,{y}_{t-1}\},\end{eqnarray*}

which is also assumed to belong to the exponential family of statistical distributions. In this context, the past data conditional PDF similar to Eq. (1) is expressed as

\begin{eqnarray*}{f}_{{Y}_{t}{|}{\mathbf{H}}_{t}}({y}_{t};{\vartheta }_{t},\varphi )=\exp \nolimits \left( \frac{{y}_{t}{\vartheta }_{t}-b({\vartheta }_{t})}{\varphi } +c({y}_{t},\varphi ) \right) , {y}_{t}\in {R}_{{Y}_{t}}. \end{eqnarray*}

Note that here the canonical parameter *ϑ*_*t*_ and values of covariates ***x***_*t*_ depend on a temporal sequence, while the parameter *φ*,  remains constant and independent of the time sequence. In this conditions, we denote the conditional mean and variance of *Y*_*t*_ given **H**_*t*_ by *μ*_*t*_ = E(*Y*_*t*_|**H**_*t*_) = *b*′(*ϑ*_*t*_) and Var(*Y*_*t*_|**H**_*t*_) = *φb*^′′^(*ϑ*_*t*_), for *t* = 1, …, *n*, respectively. *g*(*μ*_*t*_) can be expressed as a GLARMA model of *p* and *q* orders. This model is denoted by GLARMA(*p*, *q*), where

(4) \begin{eqnarray*}{\eta }_{t}=g({\mu }_{t})={\mathbi{x}}_{t}^{\top }\beta +\sum _{h=1}^{p}{\phi }_{h}(g({y}_{t-h})-{\mathbi{x}}_{t-h}^{\top }\beta )+\sum _{j=1}^{q}{\lambda }_{j}(g({y}_{t-j})-{\eta }_{t-j}).\end{eqnarray*}

In this model *ϕ*_*h*_ and *λ*_*j*_ correspond to the *h*th and *j*th components of an ARMA(*p*, *q*) model, related to the autoregressive and moving average components, respectively. *β* are the regressors, related with known values of *r* covariates, depending over time, denoted by ***x***_*t*_ = (*x*_0_, *x*_1*t*_, …, *x*_*rt*_)^⊤^, with *x*_0_ = 1. The link function *η*_*t*_ = *g*(*μ*_*t*_) of the GLARMA model given in Eq. (4) can any as the identity function inverse or logarithmic (log) functions (allowing to consider non-linear associations). In this model the variance is assumed to be constant over time. In the case of the identity link function, we have that

(5) \begin{eqnarray*}{\mu }_{t}={\mathbi{x}}_{t}^{\top }\beta +\sum _{h=1}^{p}{\phi }_{j}({y}_{t-h}-{\mathbi{x}}_{t-h}^{\top }\beta )+\sum _{j=1}^{q}{\lambda }_{j}({y}_{t-j}-{\mu }_{t-j}).\end{eqnarray*}

The above models can be extended to GSARIMA (*p*, *d*, *q*) × (*P*, *D*, *Q*)_*s*_ analogues by including seasonality (*S*) and differentiation (*D*) components as follows:

\begin{eqnarray*}g({\mu }_{t})& =& \Phi (L)(1-L)^{d}(1-{L}^{s})^{D}{\Phi }^{P}(L)({\mathbi{x}}_{t}^{\top }\beta -g({y}_{t}))+g({y}_{t})-{\Lambda }^{Q}(L)\Lambda ({L}^{s})(g({y}_{t})\nonumber\\\displaystyle & & -g({\mu }_{t}))+g({y}_{t})-g({\mu }_{t}), \end{eqnarray*}

where *s* is the length of the period (*s* =12 for monthly data with an annual cycle), ${\Phi }^{P}({L}^{s})=1-{\phi }_{1}^{P}{L}^{s}-\cdots -{\phi }_{p}^{P}{L}^{{s}^{P}},\Lambda ({L}^{s})^{Q}=1-{\lambda }_{1}^{Q}{L}^{s}-\cdots -{\phi }_{q}^{Q}{L}^{{s}^{Q}}.$

#### Parameter estimation

Considering *n* realizations of *Y*_*t*_, for *t* = 1, …, *n*, *y*_1_, …, *y*_*n*_, the likelihood function corresponds to the product of multiple conditional PDFs of *Y*_*t*_ given the past observations **H**_*t*_. In this context, considering *θ* = (*β*^⊤^, *ϑ*_*t*_, *φ*, *ϕ*^⊤^, *λ*^⊤^)^⊤^ as the vector of model parameters to be estimated, the associated log-likelihood function for *θ* is expressed by

(6) \begin{eqnarray*}\ell (\theta )=\sum _{t=1}^{n}\log \nolimits \left( {f}_{{Y}_{t}{|}{\mathbf{H}}_{t}}({y}_{t};\theta ) \right) .\end{eqnarray*}

The ML estimate of *θ*, $\widehat{\theta }$, are obtained from the derivation of Eq. (6) respect each parameter *β*, *ϑ*_*t*_, *φ*, *ϕ* and *λ*. The statistical inference of *θ* is based on the asymptotic normality of the ML estimator $\widehat{\theta }$.

#### Model checking and diagnostic

The random quantile (RQ) residual is used to diagnose the adequacy of the GLSARMA or GSARMA models to the data. RQ residual is mathematically defined as *r*_*t*_ and can be calculated by:

(7) \begin{eqnarray*}{r}_{t}={\Phi }^{-1}({F}_{{Y}_{t}{|}{\mathbf{H}}_{t}}({y}_{t};\widehat{\theta })),\end{eqnarray*}

where *F*_*Y*_*t*_|**H**_*t*__ is the cumulative distribution function (CDF) of *Y*_*t*_ conditional to past data, $\widehat{\theta }$ is the ML estimate of *θ*, and Φ^−1^ is the inverse CDF of a standard normal distribution. RQ residual follows the standard normal distribution. For more details about this residual, see Dunn & Smyth (1996).

To properly define a GSARIMA or GLARMA model, each competing model must be compared based on different combinations of order *p*, *q*. The global deviation (GD) is used as an indicator to compare these models. GD is −2 times the logarithmic probability ratio of the reduced model (in our case a GLM) and the complete model (in our case the GLARMA or GSARIMA model). To select the best competing model, the Akaike (AIC) and Bayesian (BIC) information criteria can be used. The expressions of AIC and BIC correspond to:

\begin{eqnarray*}\text{AIC}& =& -2\ell (\widehat{\theta })+2m, \end{eqnarray*}

\begin{eqnarray*}\text{BIC}& =& -2\ell (\widehat{\theta })+m\log \nolimits (n), \end{eqnarray*}

with $\ell (\widehat{\theta })$ being the log-likelihood function evaluated at $\theta =\widehat{\theta }$ and *n*, *m* being the sample size and number of model parameters, respectively. A smaller AIC or BIC indicates a better model. For more details on GD, AIC and BIC, see Stasinopoulos & Rigby (2007).

### Density forecast

The density forecast (DF) technique aims to assess predictive performance outside the sample. This technique consists of dividing the original sample of data into a training set (2/3 of the first data) that is used to estimate the parameters, and then evaluate the performance outside the sample with the third rest of the data. That is, an out-of-sample test. These out-of-sample tests should be considered in the model validation process, see details in Rojas (2016). A probability integral transform (PIT) is the cumulative probability evaluated at the actual, realized value of the target variable. It measures the likelihood of observing a value less than the actual realized value, where the probability is measured by the DF. The PIT is uniform, independent and identically distributed if the density forecast is correctly specified.

#### Forecast with GSARIMA or GLARMA models

Forecasts using GSARIMA or GLARMA models may be carried out in an analogous manner to GARCH models; see Tsay (2009). Thus, based on GSARIMA or GLARMA models, and supposing that the forecast origin is *j* = *n* and its horizon is *h*, we have the *h*-step ahead forecast is obtained from *y*_*n*+*h*_, with initial prediction or fitted value ${\hat {y}}_{n}$ at the origin *n* and forecast error ${e}_{n}(h)={y}_{n+h}-{\hat {y}}_{n}(h)$, for *h* ≥ 1.

### GSARIMA Forecast model for prevention of foodborne outbreaks by non-typhoidal salmonellosis

We adapted our methodological framework from Maëlle, Dirk & Michael (2014). In this context, we considered an a-head time series based on fitted values of GSARIMA to alert infectious outbreaks, whose can be represented for multivariate forecast time series of counts. Denote the counts as *y*_*it*_;  *i* = 1; ⋯; *m*; *t* = *n* + 1; ⋯; *n* + *h*, where *n* + *h* is the length of the forecast time series, whose begin at time *n* + 1 and *m* is the number of entities, e.g., geographical regions, hospitals or age groups, being monitored. In the context of a forecast model for future disease outbreaks, it is essential to detect future changes in the process occurring at an unknown time *τ*. As noted by Sonesson & Bock (2003), this change can be a step increase of the counts of future cases or a more gradual change. Based on the possibility of such change, for each future time *t* we want to differentiate between the two states in-control and out-of-control. At any timepoint *t*_0_ ≥ *n* + 1 the available information -i.e., fitted values counts - is defined as ${\hat {\mathbf{y}}}_{{t}_{0}}=\{{\hat {y}}_{t}:t\leq {t}_{0}\}.$ Detection is based on a statistic *m*(⋅) with a resulting alarm time ${T}_{A}=\min \{{t}_{0}\geq 1:m({\hat {\mathbf{y}}}_{{t}_{0}})\gt a\}$ where *a* is a known threshold. The functions for outbreaks detection use fitted values to estimate **y**_*t*_0__,  and compare it to the threshold *a*, above which, the current count can be considered as suspicious and, therefore, doomed as out-of-control. Then, based on Farrington et al. (1996) we designed a forecast model of outbreaks that uses the function of an algorithm called algo.farrington of survillance a package, in R software, see Höhle & Riebler (2005). R is a non-commercial and open source software for statistical analyses and plotting, which can be obtained from http://www.r-project.org. We modified this function that summarize the Farrington et al. (1996) algorithm, to take a range of *h*-step ahead forecast values of the number of counts of infection reports. This *h*-step ahead forecast values are obtained from the multivariate time series GSARIMA forecast model show in ‘Forecast with GSARIMA or GLARMA models’. For each time ahead, we use a GSARIMA simulated time series to set the number of counts in the same periods as a comparison. To obtain GSARIMA simulated time series we take command garmsim of gsarima package in R software, see Briët, Amerasinghe & Vounatsou (2013). This command requires an autoregressive representation obtained by arrep function of same package. The estimated parameters from the time series of the observed data, necessary to define the autoregressive representation, can be estimated by glarma function of the same name package in R software, see Dunsmuir (2015). Then, this is compared to the forecast values number of counts. If the forecast value is above a specific quantile of the prediction interval given by simulated time series, then an alarm is raised, see Algorithm 1.

## Results

### Simulation study

The new methodology proposed in this paper is studied using a Montecarlo (MC) simulation study.

The GSARIMA forecast is simulated by using the autoregressive representation method, which is implemented in R, using a package named gsarima. This package contains methods for the generation of random numbers with univariate structure of time series, which is expandable to a multivariate response. This package has functions that allow the generation of generalized time series for a set of statistical counting distributions that belong to the exponential family. We focused on the negative binomial distribution (NBI), given its common use in this type of modeling, due to its properties of accumulation of counts.

We generated 5,000 scenarios of simulation, establishing different conditions given by a set of parameters in each scenario, in order to verify if the amount of alarms generated varies according to the configuration of the scenarios. For example, scenarios with a positive or negative trend must have values of *d* > 0 and / or *D* > 0, and positive or negative autoregressive coefficients, respectively. In turn, scenarios with coefficients of positive moving average reveal upward trends, and vice versa. To generate these 5,000 scenarios, we used the following indicators with the mentioned distribution, selected following Briët, Amerasinghe & Vounatsou (2013):

### Statistical parameters of NBI statistical distribution

• dispersion parameter: {2,4}
• mean: {7,10}

### GSARIMA parameters

• autoregressive parameter: *p* ∼ *U*(−1, 1)
• moving average parameter: *q* ∼ *U*(−1, 1)
• seasonal autoregressive parameter: *P* ∼ *U*(−1, 1)
• seasonal moving average parameter: *Q* ∼ *U*(−1, 1)
• integration of time series parameter: *d* = {2, 1, 0}
• integration of seasonal time series parameter: *D* = {2, 1, 0}
• covariate: *x* ∼ *U*(0, 1)
• regressor coefficient: *β* = 0.7
• intercept: *β*_0_ = 1

Using these parameters, we simulated 5,000 predicted GSARIMA time series of 156 weeks, each with a frequency of 52 weeks/years (total of 3 years). In each scenario, the generated outbreak alarms were recorded following the Farrington algorithm adapted to our methodology, see Algorithm 1. To verify the differences in the quantities of alarms, in the different generated scenarios, we constructed the classification shown in Table 1, according to the parameters of our Monte Carlo simulation study.

**Table 1:** Classification for Monte Carlo study.

Type of parameter	Condition	Denomination	Condition	Denomination
autoregressive parameter	*p* < 0	negative ar	*p* > 0	positive ar
moving average parameter	*q* < 0	negative ma	*q* > 0	positive ma
seasonal autoregressive parameter	*P* < 0	negative Sar	*P* > 0	positive Sar
seasonal moving average parameter	*Q* < 0	negative Sma	*Q* > 0	positive Sma
integration of time series parameter	*d* = {1, 2}	with integration	*d* = 0	without integration
integration of seasonal time series parameter	*D* = {1, 2}	with seasonal integration	*D* = 0	without seasonal integration
dispersion parameter	2	Low dispersion	4	High dispersion
mean parameter	7	Low mean	10	High mean

**Figure 1:**
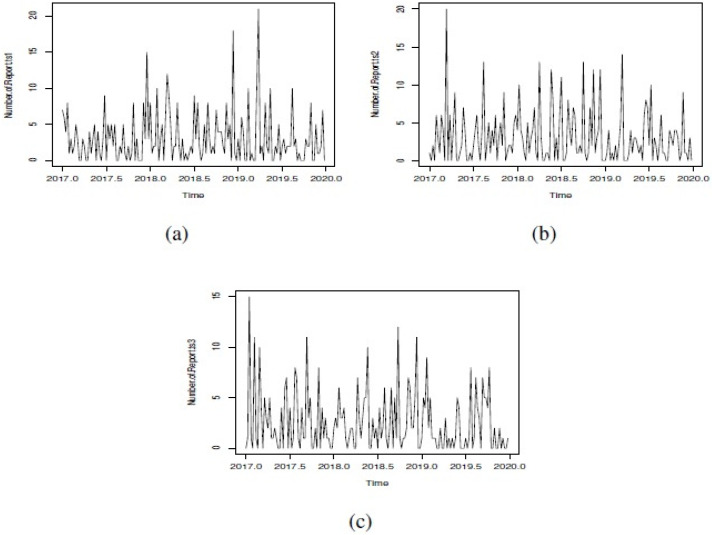
Simulated GSARIMA: (A) ts1, (B) ts2 and (C) ts3.

**Figure 2:**
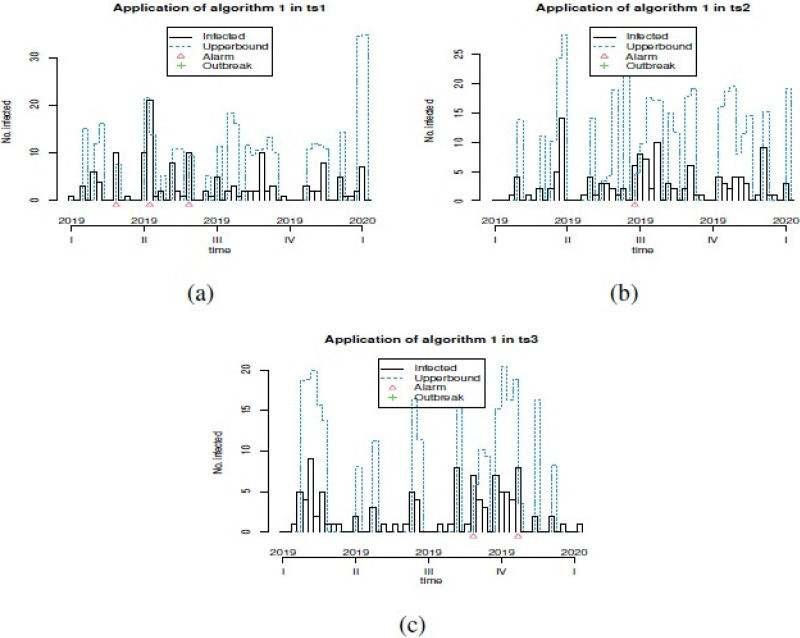
Plot of generation of alarms for prevention of outbreaks in forecast GSARIMA: (A) ts1, (B) ts2 and (C) ts3.

In order to show the different options that we can explore in our simulation study, we showed only three examples of simulated GSARIMA time series (ts) of length 156 week (3 years), using the approach mentioned in ‘GSARIMA Forecast model for prevention of foodborne outbreaks by non-typhoidal salmonellosis’. The parameters of the simulated GSARIMA ts were: *ts*1 = {*p* > 0, *q* > 0, *P* < 0, *Q* < 0, *d* = 0, *D* = 2 }, *ts*2 = {*p* > 0, *q* > 0, *P* < 0, *Q* > 0, *d* = 0, *D* = 0 }, *ts*3 = {*p* < 0, *q* > 0, *P* > 0, *Q* > 0, *d* = 1, *D* = 0}. All these simulated GSARIMA ts were made considering high dispersion and high mean parameters, and can be examined in Fig. 1. On the abscissa axis, the time is shown in years divided weekly, where for example 2018.5 means the 26th week of the year 2018 (which marks the middle of the year 2018). In Fig. 2 the upperbound is shown as a dashed line (this band shows four-week aggregated data), while the alarms -timepoints where the upperbound has been exceeded- is shown as triangles. This date was obtained by the application of Algorithm 1 to generation of alarms for prevention of outbreaks in forecast GSARIMA ts1,ts2 and ts3.

We used the Kruskal-Wallis (KW) test to compare medians of indicators related to the grouped scenario denominations according to the denominations shown in Table 1. Results of comparative of mean/median, value of statistic KW and its *p*-value by grouped scenario denominations are showed in Table 2 .

### Case study

In order to validate our model, we performed a case study based on data reports of **Salmonella enterica** serovar Enteriditis cases in Sydney, Australia (2014–2016). These data were weekly collected during three years (2014–2016) by the National Notifiable Diseases Surveillance System (NNDSS) from the Health Department of the Australian Government.

**Figure 3:**
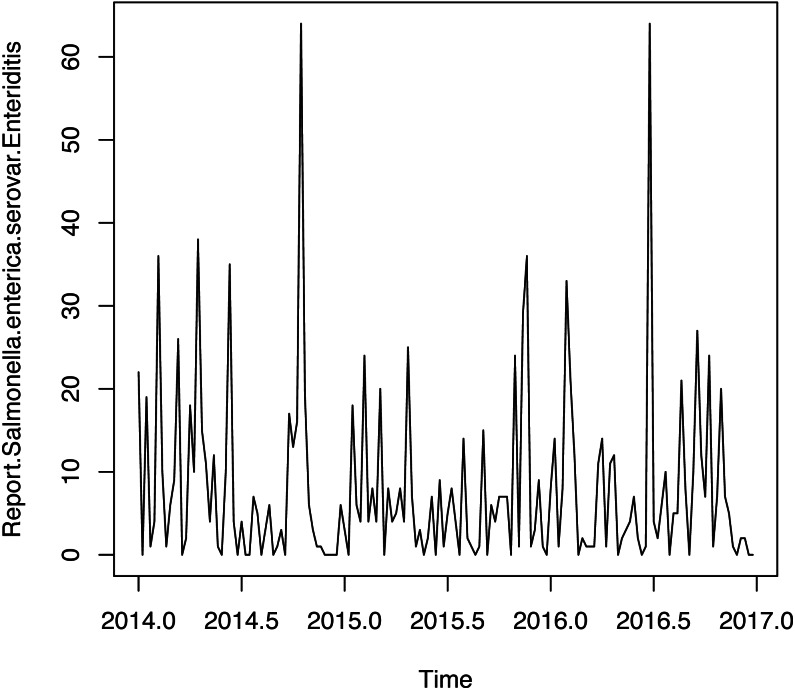
Time series weekly of number of reports of *Salmonella enterica* serovar Enteriditis cases.

**Figure 4:**
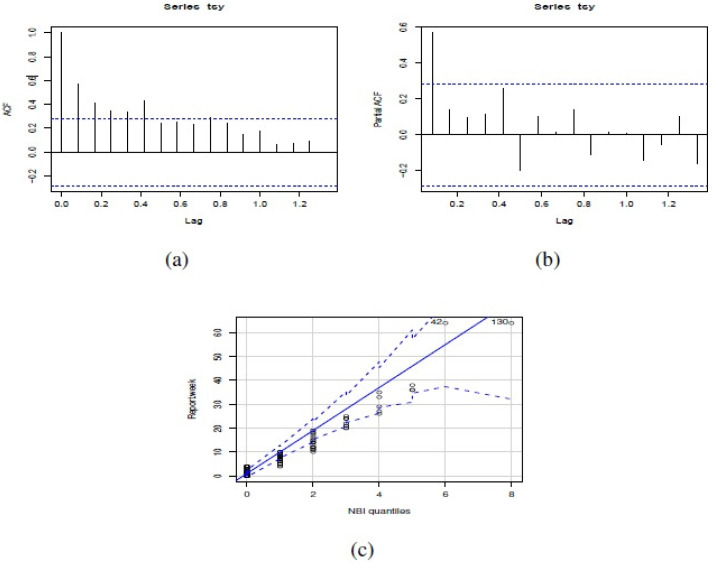
(A) ACF, (B) PACF and (C) NBII QQ-plot of the number of actual reports of *Salmonella enterica* serovar Enteriditis cases of the dataset in study.

Figure 3 shows weekly time series of number of reports of **Salmonella enterica** serovar Enteriditis cases, whereas Fig. 4 presents (a) Autocorrelation function (ACF), (b) Partial Autocorrelation function (PACF) and (c) Negative binomial (NBII) Quantil-Quantil (QQ)-plots of the variable under study (weekly time series of number of reports of **Salmonella enterica** serovar Enteriditis cases), respectively. ACF and PACF show measures of the correlation between the observations of a time series separated by *k* lag time units, in this case, *k* = 1 week. In our case, we have autocorrelation in several weeks of lag, whose are shown on the lines that exceed the confidence intervals shown by the scored lines. QQ plot indicates the comparison between theoretical quantiles (given by the proposed statistical distribution, NBII) and empirical data of the variables under study, whose all appear within the confidence intervals shown by the scored lines. Note that these figures show that the probability distribution of these variables are NBII but not independent and identically distributed. The adjusted forecast to deal with a zero inflation is covered by the proposed statistical distribution, of a negative binomial type, which is capable of admitting data at zero, assigning to this value a probability distribution according to its historical and temporal frequency. To confirm that the original time series is stationary, we applied Augmented Dickey-Fuller Test, obtaining a statistics Dickey-Fuller = −4.0722, Lag order = 3, *p*-value = 0.0144 for alternative hypothesis of time series with stationarity.

**Table 2:** Results of MC simulation study.

Type of parameter	Denomination	Mean/Median	Denomination	Mean/Median	KW	*p*-value
autoregressive parameter	negative ar	2/1.73	positive ar	2/1.87	1.7158	0.1902
moving average parameter	negative ma	2/1.74	positive ma	2/1.85	9.9086	0.0016
seasonal autoregressive parameter	negative Sar	2/1.76	positive Sar	2/1.83	1.818	0.1775
seasonal moving average parameter	negative Sma	2/1.72	positive Sma	2/1.87	17.834	0.00002
integration of time series parameter	with integration	2/1.75	without integration	2/2.12	66.72	3.129e−16
integration of seasonal time series parameter	with seasonal integration	2/1.74	without seasonal integration	2/2.1	62.122	3.229e−15
dispersion parameter	Low dispersion	2/2.09	High dispersion	1/1.37	409.68	<¡ 2.2e−16
mean parameter	Low mean	2/1.8	High mean	1/1.45	2.4248	0.1194

In this study we explored the relationship between the number of reports of **Salmonella enterica** serovar Enteriditis cases and different covariates obtained for the same time periods and city mentioned above. The consumption of undercooked eggs is known to be a risk factor for non-typhoid salmonellosis (Martelli & Davies, 2012) and infectious outbreaks caused by *Salmonella enterica* serovar Enteritidis have been widely reported worldwide (Pijnacker et al., 2019; Jiang et al., 2020; Muvhali et al., 2017; Rizzo, 2006). Additionally, environmental factors like temperature and humidity were included in this study. High temperatures have been previously correlated to an increased incidence of *Salmonella* infections (Akil, Ahmad & Reddy, 2014; Kovats et al., 2004), while humidity has been positively associated to *Salmonella* infections (Kim et al., 2015).

**Figure 5:**
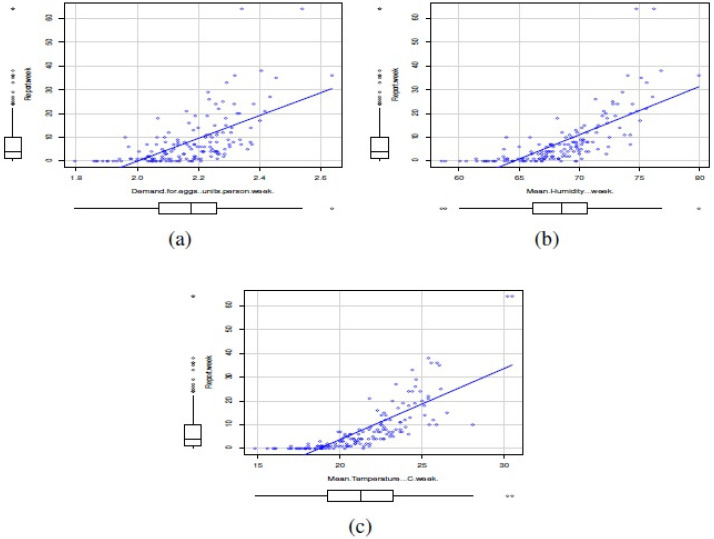
Scatterplots between number of reports of *Salmonella enterica* serovar Enteriditis as response variable and covariates: (A) weekly demand for eggs (units/person*week), (B) mean 3 pm relative humidity (%/week), and (C) mean maximum temperature per week (°C/week).

In this study, the covariates corresponded to the climatic variables of mean maximum temperature per week (°C/week), mean 3pm relative humidity (%/week), as well as the weekly demand for eggs (units/person*week). The climatological data were collected from the Bureau of Meteorology of the Australian Government, while egg consumption data were collected from the Euromonitor Agency. Figure 5 shows scatterplots between number of reports of **Salmonella enterica** serovar Enteriditis as response variable and the mentioned covariates. Note that, in general, the relationships between reports of **Salmonella enterica** serovar Enteriditis as response variable and the mentioned covariates are linear and positives. The covariates seemed to have a symmetric distribution.

We assumed a GSARIMA(*p*, *d*, *q*) model, with *p* = {0, 1, 2}, *d* = 0 and *q* = {0, 1} using a NBI statistical distribution for the number of reports de **Salmonella enterica** serovar Enteriditis cases and considered the covariates described with different link functions for the mean response. In order to select the best GSARIMA model, we used AIC, BIC and GD, whose are reported on Table 3.

From Table 3, note that the smallest AIC, BIC and GD correspond to the GSARIMA(2.0,0) model with an identity link function, which is equivalent to a GLARMA model (2.0). In order to model the response variable of number of reports of **Salmonella enterica** serovar Enteriditis (*Y*), we propose the GLARMA model given by

(8) \begin{eqnarray*}\text{E}({Y}_{j})={\mu }_{j}={\beta }_{0}+{\beta }_{1}{x}_{j}+{\phi }_{1} \left( {y}_{j-1}-{\beta }_{0}-{\beta }_{1}{x}_{j-1} \right) +{\phi }_{2} \left( {y}_{j-2}-{\beta }_{0}-{\beta }_{1}{x}_{j-2} \right) ,\end{eqnarray*}

where *β*_0_ and *β*_1_ are the regression coefficients, *x*_*j*_ is the value of the covariate vector *X* = *x*_1_, *x*_2_, *x*_3_, where *x*_1_ = Mean Maximal Temperature (° C/week), *x*_2_ = Mean 3pm Relative Humidity (%/week), *x*_3_ = weekly demand for eggs (units/person*week) and *ϕ*_1_, *ϕ*_2_ are the autoregressive coefficients. We fit the GLARMA model by using the command garmaFit. The maximum likelihood estimates of the parameters of the model given in Eq. (8), with approximate estimated standard errors in parenthesis, are: ${\widehat{\beta }}_{0}=-15.28(0.86)$, ${\widehat{\beta }}_{1}=\{{\widehat{\beta }}_{{x}_{1}}=0.25(0.019),{\widehat{\beta }}_{{x}_{2}}=0.19(0.02),{\widehat{\beta }}_{{x}_{3}}=0.95(0.58)\}$, ${\widehat{\phi }}_{1}=-0.021(0.045)$, ${\widehat{\phi }}_{2}=-0.014(0.045)$ and $(\widehat{\text{Var}}({Y}_{t}{|}{\mathbf{H}}_{t}))^{1/2}=(\widehat{\varphi }({\mu }_{t}))^{1/2}=0.041(0.016)$. All coefficients are significant at 10%. The coefficient of determination (*R*^2^) of this model is 0.88. This indicates that the variables considered in the model (*y*_*t*−1_, *y*_*t*−2_, *x*_1_, *x*_2_, *x*_3_) explain 88% of the variations of the response variable (*y*). In this model not all explanatory variables contribute the same in determining the variation of the response variable. For example, extracting the variable *x*_3_ from the model *R*^2^ remains almost unchanged (0.87), while extracting *x*_3_ and *x*_2_, *R*^2^ decreases to 0.73, leaving only the lags *y*_*t*−1_, *y*_*t*−2_ explaining the response variable *y*, *R*^2^ decreases to just 0.09. Then, prediction model can be expressed as

\begin{eqnarray*}{\widehat{\mu }}_{j+1}& =& -15.28+0.25{x}_{{1}_{j+1}}+0.19{x}_{{2}_{j+1}}+0.95{x}_{{3}_{j+1}}\nonumber\\\displaystyle & & -0.021({\widehat{y}}_{j}-(-15.28+0.25{x}_{{1}_{j}}+0.19{x}_{{2}_{j}}+0.95{x}_{{3}_{j}}))+\nonumber\\\displaystyle & & 0.041({\widehat{y}}_{j-1}-(-15.28+0.25{x}_{{1}_{j-1}}+0.19{x}_{{2}_{j-1}}+0.95{x}_{{3}_{j-1}})). \end{eqnarray*}

**Figure 6:**
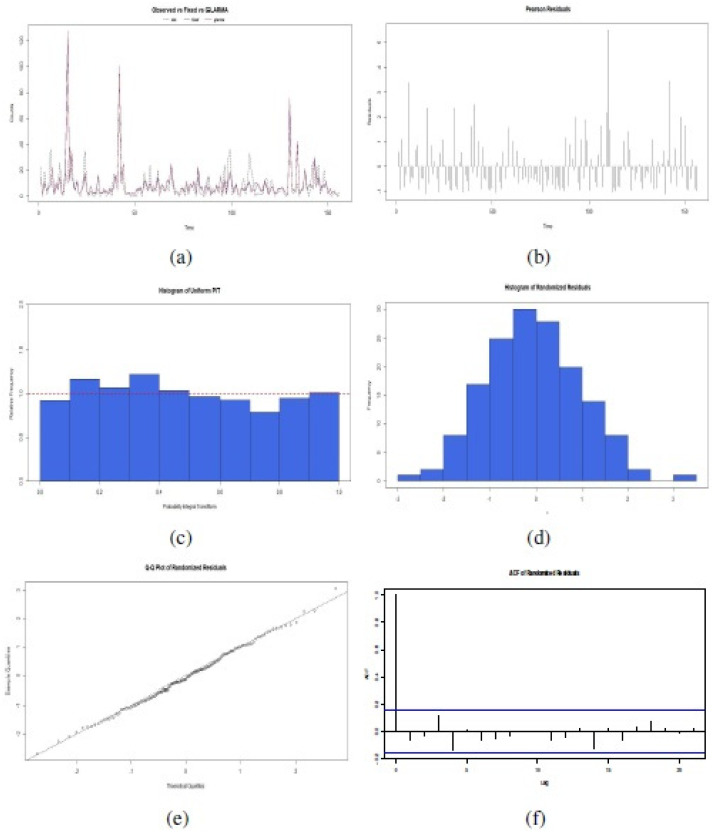
Confirmatory plot analysis of the fit of the proposed GLARMA model for the response variable in the data set of the illustrative study: (A) Observed time series related to fixed effect of GLM estimation or GLARMA estimation; (B) Pearson Residuals; (C) Histogram of Uniform PIT; (D) Histogram of Randomized Residuals normalized, (E) QQ Plots for randomized residuals of a fitted GLARMA ; and (F) Plot of the ACF of the residuals (source: authors).

To confirm the correct fit of this proposed GLARMA model, six plots were examined in Fig. 6: (a) Observed time series related to fixed effect of GLM estimation or GLARMA estimation;, (b) Disposition of Pearson Residuals in the time; (c) Histogram of Uniform PIT, (d) Histogram of Randomized Residuals normalized (randomized for a discrete response distribution); (e) Quantile-Quantile (QQ) Plots for randomized residuals of a fitted GLARMA object; and (f) Plot of the ACF of the residuals. These results indicated that is possible to apply a GLARMA model to original data, considering the covariates and logarithmic link function to the mean of response variable in each time of its realization. The selection of the best GLARMA model order is according Akaike criteria. Addiotonally, we also checked Box–Pierce test type Ljung to corroborate aleatory disposition to 2 lags with statistic *χ*^2^ = 1.18, degree of freedom (df) = 2, and *p*-value = 0.582, and normal distributions of the randomized residuals of the model, with Shapiro–Wilk normality test obtained statistic *W* = 0.9874, *p*-value = 0.9321. The forecast PDF serves to simulate future scenarios of number of reports of **Salmonella enterica** serovar Enteriditis cases.

**Table 3:** Criterion and GD for different GSARIMA models with actual data of the number of reports of *Salmonella enterica* serovar Enteriditis cases.

Information	GSARIMA(0,0,0)		GSARIMA(1,0,0)		GSARIMA(0,0,1)		GSARIMA(1,0,1)		GSARIMA(2,0,0)		GSARIMA(2,0,1)	
criterion	Identity	Log	Identity	Log	Identity	Log	Identity	Log	Identity	Log	Identity	Log
AIC	691.56	693.61	665.67	657.32	657.12	659.29	653.22	651.06	646.73	869.17	644.65	867.33
BIC	694.34	697.23	676.98	664.12	669.44	668.64	656.84	660.42	657.96	880.40	656.43	878.14
GD	683.76	683.22	657.94	650.42	648.55	648.29	634.93	641.06	634.73	857.17	633.12	856.72

**Figure 7:**
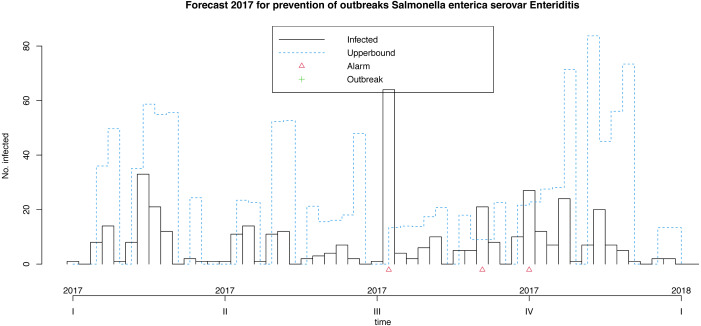
Model for the prevention of foodborne outbreaks produced by *Salmonella enterica* serovar Enteriditis to the illustrative response variable in the dataset in study (source: authors).

Following Algorithm 1 we can apply our model for prevention of future foodborne non-typhoidal salmonellosis outbreaks considered the above mentioned forecast covariates projected for the next year. These weeks results are shown in Fig. 7. Our model makes it possible to predict 3 alarms in the third quarter of next year, given the expected weather and egg consumption conditions. Remember that upperbound showed as a dashed line shows four-week aggregated data. In this case, the model does not forecast outbreaks.

## Discussion

In this work, we developed a forecast model for the prevention of foodborne outbreaks due to non-typhoidal salmonellosis, which is useful for alerting future infectious outbreaks related to the intake of foods considered of risk.

We validated our alert model for infectious outbreaks related to food consumption and weather conditions, based on real time series data of reports of **Salmonella enterica** serovar Enteriditis infections in Australia, which uses food and weather conditions as predictive variables. Through a very flexible statistical treatment granted by GSARIMA, which uses covariates and can adapt to a varied class of statistical counting distributions that can have different degrees of asymmetry as well as temporal effects of seasonality, it was possible to predict the adjusted value with high precision.

We analyzed the conceptual antecedents and the theoretical foundations that lead to the processes of our research modeling, which resulted useful for the implementation and applications of the science of administration in the field of epidemiology. Through this model, we intend to contribute with a useful tool for public health decision taking, that can accurately alert foodborne outbreaks of non-typhoidal salmonellosis. The ability to predict outbreaks of our model depends on the interaction of recognized hazards and associated conditions. Therefore, for the evaluation of the appearance of new food safety risk factors, it is necessary to study whether or not these risks are associated with outbreaks of infectious diseases, and how much they contribute to them. In this context, an application of our model could be to identify when the known or new factors can be useful or fail to predict the appearance of diseases. Failure to predict a known factor could be a signal for the emergence of a new food safety hazard.

The main limitations of our model are related to the homoscedasticity assumption of the infection report count data. Therefore, this limitation leads to a possible future topic of reserach. Apparently, this limitation could be overcome by using models of multivariate-type generalized autoregressive conditional heteroscedasticity (GARCH), whose have some attractive properties, like a greater weight on the most recent observations, but also inconveniences like an arbitrary disintegration factor that introduces subjectivity in the estimation, see Thamanukornsri & Tiensuwan (2018).

## Conclusion

We propose a forecast model for non-typhoidal salmonellosis outbreaks, a foodborne illness, which in the case of **Salmonella enterica** serovar Enteritidis is related to the consumption of eggs and climatic factors of humidity and ambient temperature.

The proposed methodology uses the modeling of infection reports by mean of GSARIMA model, which allows the use of predictive covariates. Additionally, this model can raise an alarm when a high probability of a foodborne infectious outbreak is detected, which can be useful in the surveillance and management of health care.

##  Supplemental Information

10.7717/peerj.10009/supp-1 Supplemental Information 1 Case Dataset

10.7717/peerj.10009/supp-2 Supplemental Information 2 Public Dataset of Salmonellosis in Australia 2009-2016

## Additional Information and Declarations

### Data Availability

The following information was supplied regarding data availability: Raw data is available as a Supplemental File.

## References

[ref-1] Akil L, Ahmad HA, Reddy RS (2014). Effects of climate change on Salmonella infections. Foodborne Pathogens and Disease.

[ref-2] Allen LJ (2017). A primer on stochastic epidemic models: formulation, numerical simulation, and analysis. Infectious Disease Modelling.

[ref-3] Amuakwa-Mensah F, Marbuah G, Mubanga M (2017). Climate variability and infectious diseases nexus: evidence from Sweden. Infectious Disease Modelling.

[ref-4] Ashton PM, Nair S, Peters TM, Bale JA, Powell DG, Painset A, Tewolde R, Schaefer U, Jenkins C, Dallman TJ (2016). Identification of Salmonella for public health surveillance using whole genome sequencing. PeerJ.

[ref-5] Benjamin MA, Rigby RA, Stasinopoulos DM (2003). Generalized autoregressive moving average models. Journal of the American Statistical Association.

[ref-6] Box GEP, Jenkins GM, Reinsel GC, Ljung GM (2015). Time series analysis: forecasting and control.

[ref-7] Boyen F, Haesebrouck F, Maes D, Van Immerseel F, Ducatelle R, Pasmans F (2008). Non-typhoidal Salmonella infections in pigs: a closer look at epidemiology, pathogenesis and control. Veterinary Microbiology.

[ref-8] Brauer F, Castillo-Chavez C, Feng Z (2019). Mathematical models in epidemiology.

[ref-9] Briët OJ, Amerasinghe PH, Vounatsou P (2013). Generalized seasonal autoregressive integrated moving average models for count data with application to malaria time series with low case numbers. PLOS ONE.

[ref-10] Calfa BA (2015). Data analytics methods for enterprise-wide optimization under uncertainty. Doctoral dissertation.

[ref-11] Dunn P, Smyth G (1996). Randomized quantile residuals. Journal of Computational and Graphical Statistics.

[ref-12] Dunsmuir WT (2015). Generalized linear autoregressive moving average models. Handbook of discrete-valued time series.

[ref-13] Farrington C, Andrews NJ, Beale A, Catchpole M (1996). A statistical algorithm for the early detection of outbreaks of infectious disease. Journal of the Royal Statistical Society. Series A (Statistics in Society).

[ref-14] Fischer T, Kamps U (2011). On the existence of transformations preserving the structure of order statistics in lower dimensions. Journal of Statistical Planning and Inference.

[ref-15] Gilbert K (2005). An ARIMA supply chain model. Management Science.

[ref-16] Ginsberg J, Mohebbi MH, Patel RS, Brammer L, Smolinski MS, Brilliant L (2009). Detecting influenza epidemics using search engine query data. Nature.

[ref-17] Hamlet N, Miller J, Gourlay H, Kerr J, Cunningham G (2006). Impact of a salmonella outbreak investigation in a maximum security Scottish prison. Scottish Medical Journal.

[ref-18] Höhle M, Riebler A (2005). The R package ‘surveillance’.

[ref-19] Jiang M, Zhu F, Yang C, Deng Y, Kwan PS, Li Y, Lin Y, Qiu Y, Shi X, Chen H (2020). Whole-genome analysis of *Salmonella enterica* Serovar Enteritidis isolates in outbreak linked to online food delivery, Shenzhen, China, 2018. Emerging Infectious Diseases.

[ref-20] Kim YS, Park KH, Chun HS, Choi C, Bahk GJ (2015). Correlations between climatic conditions and foodborne disease. Food Research International.

[ref-21] Konradsen F, Amerasinghe FP, Hoek WVD, Amerasinghe PH (2000). Malaria in Sri Lanka: current knowledge on transmission and control.

[ref-22] Kovats R, Edwards S, Hajat S, Armstrong B, Ebi K, Menne B (2004). The effect of temperature on food poisoning: a time-series analysis of salmonellosis in ten European countries. Epidemiology & Infection.

[ref-23] Maëlle S, Dirk S, Michael H (2014). Monitoring count time series in R: aberration detection in public health surveillance.

[ref-24] Majowicz SE, Musto J, Scallan E, Angulo FJ, Kirk M, O’brien SJ, Jones TF, Fazil A, Hoekstra RM, Enteric Disease Burden of Illness Studies IC (2010). The global burden of nontyphoidal Salmonella gastroenteritis. Clinical Infectious Diseases.

[ref-25] Martelli F, Davies RH (2012). Salmonella serovars isolated from table eggs: an overview. Food Research International.

[ref-26] McCullagh P, Nelder JA (1983). Generalized linear models.

[ref-27] Mori T (1996). Tuberculosis-epidemiology and control issues in global perspective. Journal of Epidemiology.

[ref-28] Muvhali M, Smith AM, Rakgantso AM, Keddy KH (2017). Investigation of Salmonella Enteritidis outbreaks in South Africa using multi-locus variable-number tandem-repeats analysis, 2013–2015. BMC Infectious Diseases.

[ref-29] Pijnacker R, Dallman TJ, Tijsma AS, Hawkins G, Larkin L, Kotila SM, Amore G, Amato E, Suzuki PM, Denayer S (2019). An international outbreak of *Salmonella enterica* serotype Enteritidis linked to eggs from Poland: a microbiological and epidemiological study. The Lancet Infectious Diseases.

[ref-30] Rees E, Ng V, Gachon P, Mawudeku A, McKenney D, Pedlar J, Yemshanov D, Parmely J, Knox J (2019). Risk assessment strategies for early detection and prediction of infectious disease outbreaks. Canada Communicable Disease Report.

[ref-31] Rizzo C (2006). Food-borne outbreak caused by *Salmonella enterica* serovar Enteritidis in Bari, Italy. Igiene e Sanita Pubblica.

[ref-32] Rojas F (2016). Time dependence in joint replacement to multi-products grouped. The case of hospital food service. Cogent Engineering.

[ref-33] Rojas F, Leiva V, Wanke P, Lillo C, Pascual J (2019). Modeling lot-size with time-dependent demand based on stochastic programming and case study of drug supply in Chile. PLOS ONE.

[ref-34] Santillana M, Nguyen AT, Dredze M, Paul MJ, Nsoesie EO, Brownstein JS (2015). Combining search, social media, and traditional data sources to improve influenza surveillance. PLOS Computational Biology.

[ref-35] Santillana M, Zhang DW, Althouse BM, Ayers JW (2014). What can digital disease detection learn from (an external revision to) Google Flu Trends?. American Journal of Preventive Medicine.

[ref-36] Sonesson C, Bock D (2003). A review and discussion of prospective statistical surveillance in public health. Journal of the Royal Statistical Society: Series A (Statistics in Society).

[ref-37] Stasinopoulos D, Rigby R (2007). Generalized additive models for location, scale and shape (GAMLSS). Journal of Statistical Software.

[ref-38] Thakur V, Anbanandam R (2017). Management practices and modeling the seasonal variation in health care waste: a case study of Uttarakhand, India. Journal of Modelling in Management.

[ref-39] Thamanukornsri C, Tiensuwan M (2018). Applications of box-Jenkins (Seasonal ARIMA) and GARCH models to dengue incidence in Thailand. Model Assisted Statistics and Applications.

[ref-40] Tsay R, Mills TC, Patterson K (2009). Autoregressive conditional duration models. Handbook of econometrics.

[ref-41] Yerushalmy J, Palmer CE (1959). On the methodology of investigations of etiologic factors in chronic diseases. Journal of Chronic Diseases.

